# Supplementary material for: Duoculture diminishes social interaction costs and improves growth rates of two fish species at different temperatures
Source: PLoS One. 2024 May 20;19(5):e0291886. doi: 10.1371/journal.pone.0291886 (PMC11104676; doi:10.1371/journal.pone.0291886)
Supplement: S1 File — (PDF) [file pone.0291886.s001.pdf]

Supplementary Materials

Tank No.	Group	Feed amount (g) Initial (06/17)	Remaining (06/23)	Used
4	YP I	40	22.9	17.1
1	YP II	40	25.3	14.7
15	YP III	40	27.1	12.9
3	BG I	40	19.3	20.7
16	BG II	40	19.1	20.9
13	BG III	40	23	17
2	YPBG I	40	21.9	18.1
12	YPBG II	40	21.7	18.3
14	YPBG III	40	24.1	15.9

Feed amount (g) Initial (06/23)	Remaining (07/01)	Used
50	21.7	28.3
50	30.1	19.9
50	30.6	19.4
50	23.7	26.3
50	33.6	16.4
50	29.8	20.2
50	30.2	19.8
50	26.8	23.2
50	28.6	21.4

Tank No.	Group	Feed amount (g) Initial (07/08)	Remaining (07/15)	Used
4	YP I	50	19.4	30.6
1	YP II	50	28	22
15	YP III	50	28.4	21.6
3	BG I	50	28.3	21.7
16	BG II	50	30.2	19.8
13	BG III	50	34	16
2	YPBG I	50	29.8	20.2
12	YPBG II	50	27.9	22.1
14	YPBG III	50	32.5	17.5

Feed amount (g) Initial (07/15)	Remaining (07/22)	Used
60	19.4	40.6
60	33.5	26.5
60	30.4	29.6
60	31.1	28.9
60	38.7	21.3
60	40.7	19.3
60	32.3	27.7
60	28.3	31.7
60	36.1	23.9

Tank No.	Group	Feed amount (g) Initial (07/29)	Remaining (08/05)	Used
4	YP I	70	17	53
1	YP II	70	22.9	47.1
15	YP III	70	22.5	47.5
3	BG I	70	31.3	38.7
16	BG II	70	44.2	25.8
13	BG III	70	47.2	22.8
2	YPBG I	70	28	42
12	YPBG II	70	26.8	43.2
14	YPBG III	70	35.2	34.8

Feed amount (g) Initial (08/05)	Remaining (08/12)	Used
100	19.6	80.4
80	16.7	63.3
100	31.2	68.8
80	27.2	52.8
80	48	32
80	56.5	23.5
80	20.6	59.4
80	16.6	63.4
80	34.7	45.3

Tank No.	Group	Feed amount (g) Initial (08/19)	Remaining (08/26)	Used
4	YP I	123	23.7	99.3
1	YP II	110	26.4	83.6
15	YP III	90	17.4	72.6
3	BG I	90	29.5	60.5
16	BG II	90	54.4	35.6
13	BG III	90	53	37
2	YPBG I	110	17	93
12	YPBG II	90	28.6	61.4
14	YPBG III	90	29.6	60.4

Feed amount (g) Initial (08/26)	Remaining (09/02)	Used
150	29.2	120.8
130	24.9	105.1
130	52.8	77.2
80	20.1	59.9
80	49.6	30.4
80	51.6	28.4
130	40.7	89.3
130	44	86
130	52	78

Tank No.	Group	Feed amount (g) Initial (09/09)	Remaining (09/16)	Used
4	YP I	140	56.1	83.9
1	YP II	80	25.3	54.7
15	YP III	80	27.6	52.4
3	BG I	80	45.6	34.4
16	BG II	80	58.5	21.5
13	BG III	80	53.8	26.2
2	YPBG I	80	32.3	47.7
12	YPBG II	80	21.8	58.2
14	YPBG III	80	26.2	53.8

Feed amount (g) Initial (09/16)	Remaining (09/23)	Used
130	21.9	108.1
130	41.4	88.6
130	31.1	98.9
80	47.4	32.6
80	60.3	19.7
80	55.9	24.1
130	64.8	65.2
130	45.2	84.8
130	62.5	67.5

Tank No.	Group	Feed amount (g) Initial (09/29)	Remaining (10/06)	Used
4	YP I	150	46	104
1	YP II	150	49	101
15	YP III	150	39	111
3	BG I	90	44	46
16	BG II	90	64	26
13	BG III	90	52	38
2	YPBG I	150	73	77
12	YPBG II	150	47	103
14	YPBG III	150	74	76

Feed amount (g) Initial (10/06)	Remaining (10/13)	Used
150	23	127
150	37	113
150	17	133
90	28	62
90	40	50
90	37	53
150	43	107
150	37	113
150	50	100

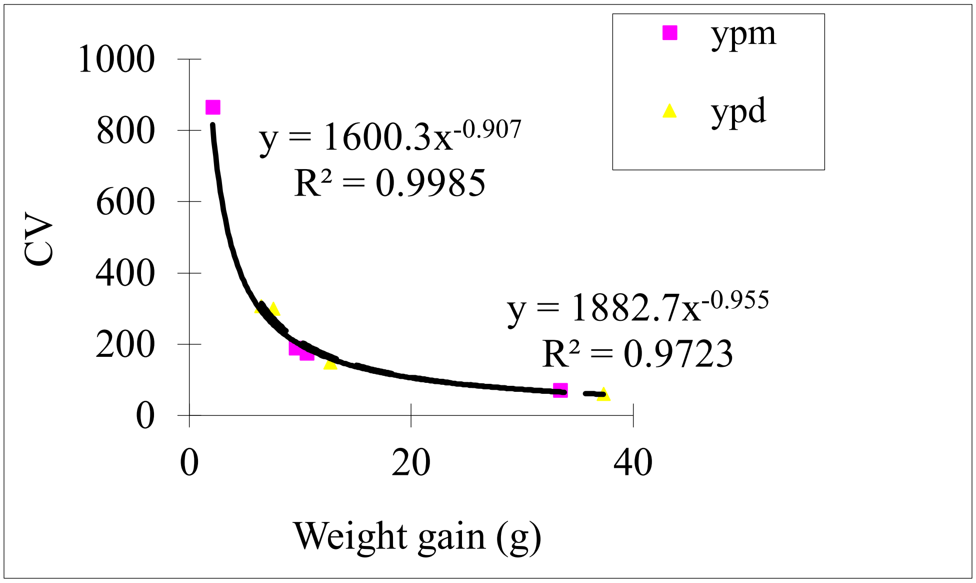
ypm
ypd
1000
800
600
400
200
0
CV
0
20
40
Weight gain (g)
y = 1600.3x-0.907
R² = 0.9985
y = 1882.7x-0.955
R² = 0.9723

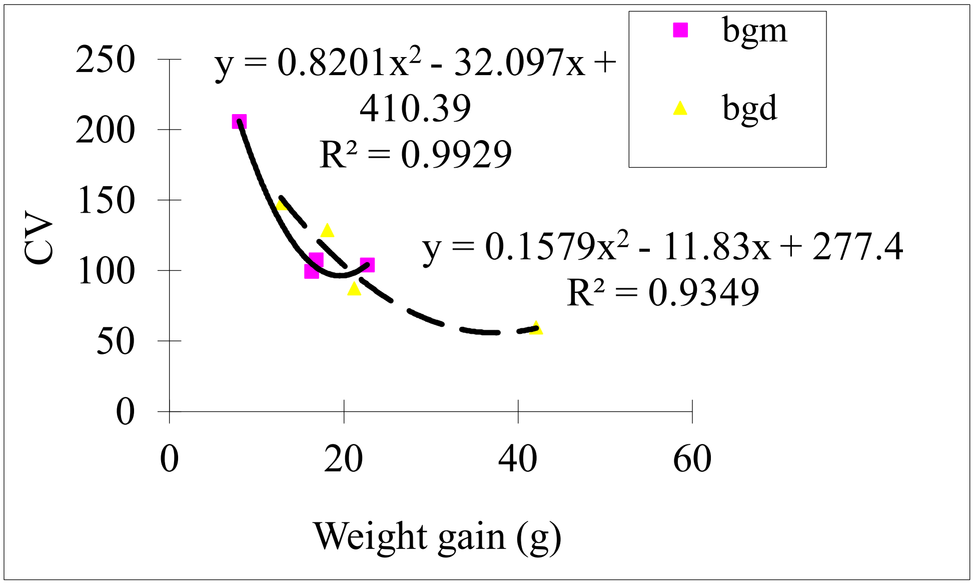
bgm
bgd
250
200
150
100
50
0
CV
0
20
40
60
Weight gain (g)
y = 0.8201x2 - 32.097x + 410.39
R² = 0.9929
y = 0.1579x2 - 11.83x + 277.4
R² = 0.9349
